# The RESISTANT study (Respiratory Muscle Training in Patients with Spinal Muscular Atrophy): study protocol for a randomized controlled trial

**DOI:** 10.1186/s12883-023-03136-3

**Published:** 2023-03-23

**Authors:** Kim Kant-Smits, Bart Bartels, Fay-Lynn Asselman, Esther S. Veldhoen, Ruben P. A. van Eijk, W. Ludo van der Pol, Erik H. J. Hulzebos

**Affiliations:** 1 Child Development and Exercise Center, Wilhelmina Children’s Hospital, University Medical Center Utrecht, Lundlaan 6, PO Box 85090, 3508 AB Utrecht, The Netherlands; 2 Department of Neurology and Neurosurgery, UMC Utrecht Brain Center, University Medical Center Utrecht, Utrecht, The Netherlands; 3 Department of Pediatric Intensive Care, University Medical Centre Utrecht, Utrecht, The Netherlands; 4 Biostatistics & Research Support, Julius Center for Health Sciences and Primary Care, University Medical Center Utrecht, Utrecht, The Netherlands

**Keywords:** Spinal muscular atrophy, Inspiratory muscle training, Expiratory muscle training, Maximum inspiratory mouth pressure, Maximum expiratory mouth pressure

## Abstract

**Background:**

Spinal Muscular Atrophy (SMA) is characterized by progressive and predominantly proximal and axial muscle atrophy and weakness. Respiratory muscle weakness results in impaired cough with recurrent respiratory tract infections, nocturnal hypoventilation, and may ultimately lead to fatal respiratory failure in the most severely affected patients. Treatment strategies to either slow down the decline or improve respiratory muscle function are wanting.

**Objective:**

The aim of this study is to assess the feasibility and efficacy of respiratory muscle training (RMT) in patients with SMA and respiratory muscle weakness.

**Methods:**

The effect of RMT in patients with SMA, aged ≥ 8 years with respiratory muscle weakness (maximum inspiratory mouth pressure [PImax] ≤ 80 Centimeters of Water Column [cmH2O]), will be investigated with a single blinded randomized sham-controlled trial consisting of a 4-month training period followed by an 8-month open label extension phase.

**Intervention:**

The RMT program will consist of a home-based, individualized training program involving 30-breathing cycles through an inspiratory and expiratory muscle training device. Patients will be instructed to perform 10 training sessions over 5–7 days per week. In the active training group, the inspiratory and expiratory threshold will be adjusted to perceived exertion (measured on a Borg scale). The sham-control group will initially receive RMT at the same frequency but against a constant, non-therapeutic resistance. After four months the sham-control group will undergo the same intervention as the active training group (i.e., delayed intervention). Individual adherence to the RMT protocol will be reviewed every two weeks by telephone/video call with a physiotherapist.

**Main study parameters/endpoints:**

We hypothesize that the RMT program will be feasible (good adherence and good acceptability) and improve inspiratory muscle strength (primary outcome measure) and expiratory muscle strength (key secondary outcome measure) as well as lung function, patient reported breathing difficulties, respiratory infections, and health related quality of life (additional secondary outcome measures, respectively) in patients with SMA.

**Discussion:**

RMT is expected to have positive effects on respiratory muscle strength in patients with SMA. Integrating RMT with recently introduced genetic therapies for SMA may improve respiratory muscle strength in this patient population.

**Trial registration:**

Retrospectively registered at clinicaltrial.gov: NCT05632666.

## Background

Spinal muscular atrophy (SMA) is a severe neuromuscular disease caused by a homozygous deletion of the survival motor neuron-1 (*SMN*) gene [1–4], which leads to cellular SMN protein deficiency. SMA has an incidence of about 1 in 6000–12,000 live births [5]. The main characteristic of SMA is the degeneration of alpha motor neurons in the anterior horns of the spinal cord, resulting in progressive muscle weakness of axial muscles and muscles of the arms and legs with a mild to severely reduced life expectancy in the majority of patients [6, 7]. SMA is classified into four types based on age at onset and highest acquired motor milestone [2, 4, 8–11]. In the last few years, SMN-augmenting genetic therapies have been introduced, including SMN-gene therapy (Zolgensma®) and therapies that modify SMN2-splicing (Spinraza® and Risdiplam) [12]. Efficacy studies have demonstrated, on average, improved motor function, survival, and overall muscle strength, but the respiratory outcomes vary, with most studies showing no significant improvement in lung function parameters in patients with SMA types 2 and 3 [13–16].

Respiratory problems are among the principal challenges in clinical care for patients with SMA [6]. Weakness of respiratory muscles requires daily interventions and thereby profoundly affects quality of life [8, 17–19]. Progressive decline of vital capacity and cough strength causes respiratory failure in virtually all children with SMA type 1 [20–22]. In more chronic types of SMA (type 2 and type 3), weakness or dysfunction of the respiratory musculature leads to severe respiratory complications [20–22]. These include reduced cough strength and poor secretion clearance resulting in recurrent respiratory tract infections, reduced chest wall and pulmonary compliance with restrictive lung function decline, alveolar hypoventilation, and, finally, chronic respiratory failure leading to premature death [20–22].

In healthy subjects and patients with pulmonary diseases, kyphoscoliosis, or Duchenne’s Muscular Dystrophy, respiratory muscle training (RMT) has been shown to improve respiratory muscle strength as well as endurance [23–26]. Little is known, however, about therapeutic benefits of RMT in patients with SMA. A pre-experimental study in three children with SMA showed that inspiratory muscle training was safe, feasible and acceptable and improved inspiratory muscle strength and peak inspiratory flow [27]. Importantly, none of these children had received any form of SMN-augmenting therapy that has also been shown to exert positive effects on overall muscle strength in some patients with SMA [24].

To further investigate treatment efficacy of RMT in SMA, we have designed a randomized controlled trial to study the efficacy of a 4-month home-based RMT program in patients with SMA including patients that were recently started on SMN-augmenting therapy.

## Methods

### Aim

The aim of this study is to assess the feasibility and efficacy of respiratory muscle training (RMT) in patients with SMA and respiratory muscle weakness. We hypothesize that an individualized incremental home-based RMT program will be feasible and may improve inspiratory muscle strength, expiratory muscle strength, lung function and patient reported breathing difficulties in patients with SMA.

### Study setting

We will conduct this study at the outpatient department of the Netherlands SMA center, and the Child Development and Exercise Center at the University Medical Center Utrecht (UMCU), The Netherlands. All members of the study team, consisting of physicians, physiotherapists, lung function technicians, clinical exercise physiologists and nurses, have broad experience with SMA due to the national cohort study that is carried out in this center since 2010 [28].

### Study design

**Fig. 1:**
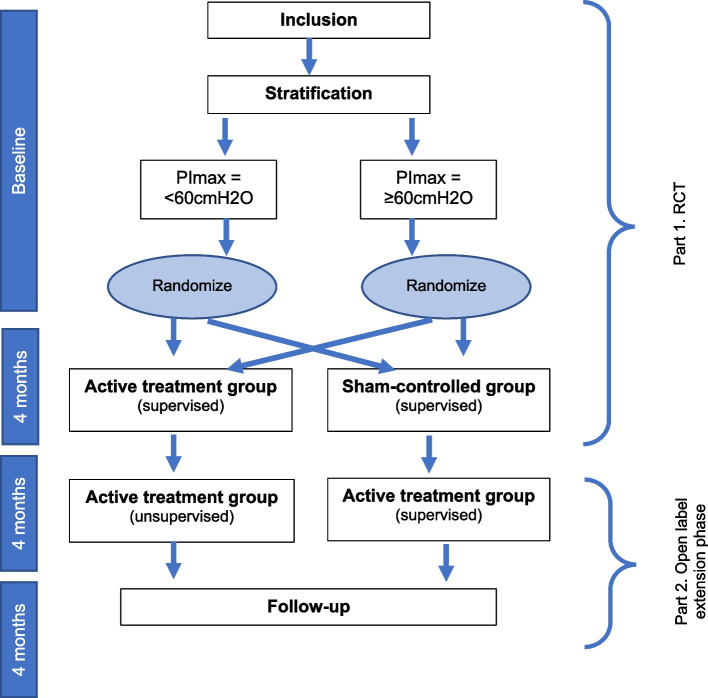
Study design

The study protocol was designed using the recommendations of the Standard Protocol Items: Recommendations for Interventional Trials (SPIRIT) guidelines [29]. The RESISTANT study is an investigator-initiated, monocenter study consisting of two parts (see Fig. 1).

#### Part 1 (0–4 months): a single blinded randomized sham-controlled trial (RCT)

In the first part of the study, we will determine the feasibility and efficacy of respiratory muscle training (RMT) in patients with SMA. The active treatment group will receive inspiratory muscle training starting at a therapeutic intensity of 30% of maximum inspiratory mouth pressure (PImax) and expiratory muscle training starting at a therapeutic intensity of 30% of maximum expiratory mouth pressure (PEmax) for 4 months [30]. The sham-control group will receive the same training protocol but with a low (10% of PImax and PEmax) non-therapeutic intensity. Both groups will receive supervision through two-weekly telephone/video calls with a physiotherapist.

Patients will be stratified prior to randomization based on PImax (group 1: PImax < 60 Centimeters of Water Column [cmH2O], group 2: PImax ≥ 60 cmH2O, 60 cmH2O was the median PImax in the group of patients used for the natural history study [31]) and then randomly allocated to the intervention or sham-control group. We will use a variable block randomization method with allocation concealment in a centralized system for randomization. The lung function analyst is blinded for treatment allocation. A data analyst (RvE) will design and sign the data analysis plan in advance. The data will be analyzed according to the analysis plan by a physiotherapist (KK) who is not blinded for treatment allocation. The physiotherapists who will perform the two-weekly telephone calls (KK and EH) are not blinded for treatment allocation. Patients will know that there are two treatment groups, and they are informed that it is not yet known which treatment is most effective.

#### Part 2 (5–12 months): open label extension phase

In the second part of the study, the sham-control group will be provided with a supervised RMT at a therapeutic intensity of 30% of PImax and PEmax and we will explore the long-term effects of RMT on the occurrence of respiratory infections, health related quality of life and feasibility in the active treatment and sham-controlled group.

Participants will visit our outpatient department every 4 months for 12 months after inclusion for assessment of primary and secondary outcome measures. This study is currently ongoing; the first participant was included on 2–2-2021. We expect study completion in the first quarter of 2023.

## Participants

### Recruitment

We will recruit patients with SMA from the Dutch national SMA registry, that contains detailed clinical data of more than 400 patients [11].

### Eligibility criteria

Patients with SMA (any type) will be invited to participate. Inclusion criteria are:

- Age ≥ 8 years;
- Respiratory muscle weakness (PImax ≤ 80 cmH2O [32]);
- Maintenance dose (≥ 2 months) Spinraza® or (≥ 2 months) Risdiplam or no treatment;
- Given oral and written informed consent when ≥ 16 years old and additional informed consent by the parents or legal representative if the participant is < 16 years old.

Exclusion criteria are:

- Inability to perform respiratory and/or lung-function testing;
- Inability to understand Dutch or English;
- A history of pneumothorax or symptomatic low cardiac output syndrome;
- Treatment period < 2 months of Spinraza® or Risdiplam.

### Sample size

Based on a previous report on inspiratory muscle training in patients with neuromuscular diseases (*n* = 27, 18 patients with Duchenne Muscular Dystrophy [DMD] and 9 patients with SMA) [17] indicating a mean improvement in PImax of 28 cmH2O difference (Standard Deviation [SD] ± 26.27), we assume a mean difference between active and sham-treated patients after 4 months of 20 cmH2O (SD 25.0). To detect this effect size with 80% power and two-sided alpha of 5%, 50 patients are needed (25 per group).

## Intervention

### Inspiratory muscle training

For the inspiratory muscle training (IMT) we use the POWERbreathe KHP2 [33]. Clinical research has shown high participant motivation and adherence to training with the POWERbreathe KHP2 thanks to the on-screen feedback [34]. Furthermore, healthcare professionals can review participant progress by tracking up to 30 of the participants training sessions which the KHP2 is able to store. This data can be scrolled through to monitor progress. The electronic, variable, tapered flow valve ensures maximum training benefit. It is easy to use, easy to clean and training improvements can be easily monitored [33].

### Expiratory muscle training

For the expiratory muscle training (EMT) we use the Threshold Inspiratory Muscle Trainer (IMT) (Philips Respironics) in reverse. Use of the Threshold Positive Expiratory Pressure (PEP) (Philips Respironics) is one method to perform EMT. However, the maximal expiratory resistance of the Threshold PEP is limited to 20 cmH20 [35]. To overcome this limitation in expiratory resistance, we chose the reverse use of the Threshold IMT [35–37]. This device contains, at its end, a valve closed by the positive pressure of a spring, which can be graded from 9 to 42 cmH2O and allows resistance changes by 1 cmH2O increments. The reverse Threshold IMT has a one-way spring-loaded valve, that closes during expiration and requires that participants exhale hard enough, to open the valve and let the air go out. This device provides constant pressure for expiratory muscle training, regardless of how quickly or slowly the participant breathes, and the optimal loading pressure can be adjusted, based upon the individual characteristics of the participant [35, 37, 38].

## Participant timeline

The study schedule is presented in Table 1. Before the first visit, participants will be recruited for enrollment by a research nurse. Patients who express interest in participating receive a patient information letter and an appointment with the physiotherapist. At the first visit (M0), the physiotherapist further determines whether patients are eligible for participation. After signing the informed consent form, participants are weighed and their length is determined, followed by lung function tests. If PImax > 80 cmH2O, participants are excluded for the study. If PImax ≤ 80 cmH2O, participants will be stratified (group 1 < 60 cmH2O, group 2 ≥ 60 cmH2O) and then randomly allocated to either the active treatment group or the sham-controlled group.

**Table 1:**
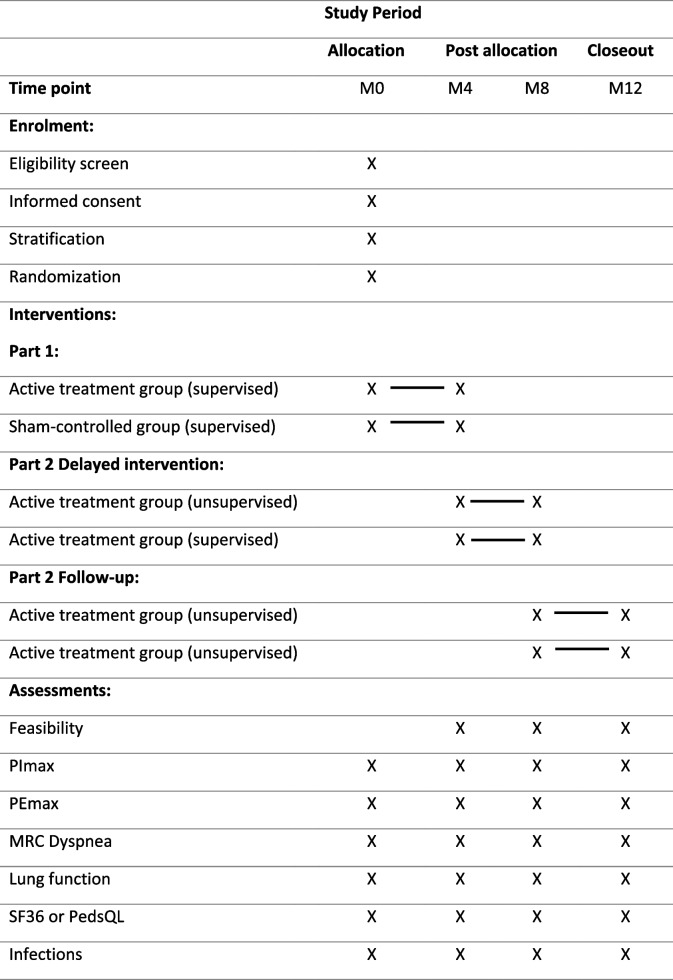
Study schedule of enrolment, interventions, and assessments

*M* month, *MRC* Medical Research Council, *PEmax* maximum expiratory mouth pressure, *PedsQL* Pediatric Quality of Life Inventory, *PImax* maximum inspiratory mouth pressure, *SF36* 36-item Short Form Health Survey

All participants (and parents) will be instructed by a trained physiotherapist on the use of both devices at the first visit (M0). Participants are instructed to aim for 10 training sessions per week, divided over 5 to 7 days. A minimum of 6 hours in between training sessions is recommended. Per training session, the participant breathes 30 times through the POWERbreathe and 30 times through the reverse Threshold IMT. If necessary, the participant may take a break, with a maximum of 60 seconds after 10 or 15 breaths. After each session they fill in a diary, which contains information about the intensity of the training and the perceived exertion (Borg scale 0–10).

In the active treatment group, the intensity of the training is set at M0 at 30% of PImax and PEmax and will be increased or decreased based on level of perceived exertion. Participants are instructed to increase the intensity with 1–5 cmH2O if they score a perceived exertion of 0–4 and decrease the intensity if they score a perceived exertion of 7–10. If they score a perceived exertion of 5 or 6, the intensity will not be adjusted. The intensity of the training in the sham-controlled group will be set at M0 at 10% of PImax and PEmax and will remain the same during the first 4 months of training. After 4 months we will provide the sham-controlled group with the same training regime as the active treatment group.

## Data collection

### Baseline measures

We will record the following baseline data: gender, age, SMA type, number of *SMN2* copies, type of SMN augmenting therapy, use of other medication, co-morbidities, ambulatory level according to the modified Hoffer classification [39], use of ventilatory support and use of Airway Clearance Techniques (ACT) (Airstacking [AS] or Mechanical insufflation-exsufflation [MI-E]).

### Outcome measures

This study investigates the feasibility and efficacy of respiratory muscle training in patients with SMA. The lung function analysts, who are blinded for treatment allocation, administer the questionnaires (health related quality of life, Medical Research Council [MRC] dyspnea and dyspnea immediately after lung function measure), perform lung function tests, and measure respiratory muscle strength, in a fixed order.

#### Feasibility

Feasibility will be determined based on adherence and acceptability. Adherence is defined as the completion rate of the estimated number of training sessions over 4 months (≥ 80% of the participants have fulfilled the prescribed treatment = good adherence). Adherence will be monitored by a patient diary, two weekly telephone- or video calls with a physiotherapist and the number of training sessions in the POWERbreathe KHP2. Acceptability is defined as the willingness to continue the training (≥ 5 = good acceptability) and will be assessed with a Borg Scale (0–10) at M4, M8 and M12 by the physiotherapist.

#### Efficacy: primary and key secondary outcome measure

##### PImax and PEmax

To measure the efficacy, we will examine changes in respiratory performance over time in both groups. Measurements of PImax and PEmax is a simple assessment of global respiratory muscle strength in a clinical setting and the test is responsive to evaluate changes within subjects. PImax and PEmax in kiloPascal (kPa) is assessed conform the European Respiratory Society/American Thoracic Society (ERS/ATS) recommendations [32]. PImax and PEmax will be converted to cmH2O by multiplying the value in kPa by 10.197. Reference values of Wilson et al. [40] will be used to calculate % of predicted.

#### Efficacy: secondary outcome measures

To additionally investigate the effect of the respiratory muscle training on daily life functioning, lung function and respiratory infections we use the following measures:

##### Health related quality of life

Health related quality of life will be measured with the 36-item Short Form Health Survey (SF36) for adults and the Pediatric Quality of Life Inventory (PedsQL) for children. The SF36 Health Survey is composed of 36 questions and standardized response choices, organized into eight multi-item scales: physical functioning (PF), role limitations due to physical health problems (RP), bodily pain (BP), general health perceptions (GH), vitality (VT), social functioning (SF), role limitations due to emotional problems (RE), and general mental health (MH). All raw scale scores are linearly converted to a 0 to 100 scale, with higher scores indicating higher levels of functioning or well-being [41]. The scores of the different scales will be summarized into a physical component summary (PCS) and a mental component summary (MCS) [42]. The PedsQL generic score scale consists of 23 items and has a child self-report format for ages 5–7, 8–12, and 13–18 years. The items are scored on a five-point Likert-scale, ranging from ‘never a problem’ to ‘almost always a problem’ (corresponding scores 100, 75, 50, 25 or 0) and are organized into four multidimensional scales: physical functioning, emotional functioning, social functioning, and school functioning and three summary scores: total scale score, physical health summary score, and psychosocial health summary score. A higher PedsQL score indicates a better quality of life [43].

##### Lung function

Lung function testing includes spirometry with measurements of upright (forced) vital capacity ([F]VC) in liters, peak expiratory flow (PEF) in liters per second, forced expiratory volume in 1 s (FEV1) in liters, peak cough flow (PCF) in liters per second, sniff nasal inspiratory pressure (SNIP) in cmH2O and mouth occlusion pressure at 100 ms during quiet breathing (P0.1) in kPa. P0.1 is intended to measure the actual central respiratory drive [44]. P0.1/PImax is the ratio between the respiratory drive and the capacity of the inspiratory muscles and have been suggested as important predictor of impending respiratory muscle fatigue (work of breathing) [44]. Lung function is assessed conform the ERS/ATS recommendations [32]. Global lung function reference equations for VC [45], FVC and FEV1 [46], PEF [47, 48], and P0.1 [44, 49] will be used to calculate % of predicted.

For the use of the reference equations, height in centimeters and weight in kilogram will be needed. Height is assessed using the ulna method [50]. This method is useful for determining the height of wheelchair bound patients and those with curvature of the spine. Weight will be measured with a passive floor lift (Maxi Move, type Arjo).

##### Patient reported breathing difficulties

Patient reported impact of breathing difficulties will be measured with the Medical Research Council (MRC) dyspnea scale. The dyspnea scale has been in use for many years for grading the effect of breathlessness on daily activities. This scale measures perceived respiratory disability. The MRC dyspnea scale is simple to administer as it allows the patients to indicate the extent to which their breathlessness affects their mobility [51, 52]. Dyspnea immediately after lung function measure and after each training session is measured with a Borg scale ranging from 0–10.

##### Respiratory infections

Respiratory infection frequency (based on the need for antibiotics and/or hospitalization) will be assessed during the two weekly telephone consultations and during each visit by the physiotherapist. In case of uncertainties the general practitioner, the neurologist or the pharmacy of the patient will be consulted.

##### Adverse Events (AEs)

All AEs that are reported spontaneously by the participant or observed by the investigator or study staff members are recorded and if necessary, appropriate measures are taken.

### Statistical analysis

Continuous variables will be expressed as means with standard deviations or medians with interquartile ranges (whichever is more appropriate), and discrete variables will be expressed as numbers with percentages. The main efficacy population will consist of all patients being randomized and analyzed according to their original treatment allocation, irrespective of actual received treatment or follow-up (intention-to-treat). The primary comparison will be the mean difference in PImax % of predicted at month 4. For the secondary outcome measures we will compare the mean difference in health-related quality of life, PEmax % of predicted, VC % of predicted, FVC % of predicted, FEV1% of predicted, PEF % of predicted, P0.1% of predicted, PCF, SNIP, P0.1/PImax and patient reported breathing difficulties. An ANCOVA model will be used to analyze the differences between groups adjusting for baseline values. Missing data in the outcomes at month 4 will be imputed by the baseline-observation-carried-forward (BOCF) approach. This will be a conservative method because we expect patients’ PImax will improve after training. For the longitudinal data, we will use a mixed model for repeated measurements including a term for visit, treatment, their interaction, and baseline PImax to account for the correlation within subjects. Similar models will be used for the secondary endpoints. We will summarize incidence of respiratory infections and other AEs by treatment group and in all treatment groups combined in frequency tables, coded according to the introductory guide Medical Dictionary for Regulatory Activities (MedDRA) version 21.0 [53].

### Data management

The following measures will be taken to assure the confidentiality and anonymity of the participants’ data or documents collected in Castor: a) each participant will be identified in an electronic database by a unique six digit code; b) the list of participant names corresponding to the codes will be stored in a separate encrypted electronic database, safeguarded by the principal investigator; c) only study investigators will have access to the databases and examine individual data or documents; d) all logins will be recorded; e) adopt strict precautions to prevent access to the data or documents by non-authorized persons; f) the handling of data and documents will comply with the General data protection regulation (GDPR) and is further described in the Data Monitoring Plan.

### Ethics, dissemination, and safety monitoring

This study is registered in the American registry for clinical studies and trials (NCT05632666; https://clinicaltrials.gov). The investigator obtains written informed consent before study participation from participants and from parents if the participant is < 16 years old.

The trial is monitored by an external independent party (Julius Clinical). Because of the negligible risk classification minimal monitoring will be necessary. All participants are insured by the sponsor in case of harm due to study participation.

The study will be conducted according to the principles of the Declaration of Helsinki, adapted 19–10-2013, and in accordance with the Medical Research Involving Human Subjects Act (WMO). The code of Conduct as agreed upon 2001 by the Dutch organization of Pediatrics will be used. The study is partly done by minors, which means that in any case of resistance the test and research protocol will be terminated. Resistance means that the participant’s behavior obviously differs from or is more excessive compared to participant’s normal behavior. The national rules of the Dutch Association of Pediatrics for protection of minor study participants, are followed during the entire study. The results of this study will be publicly disclosed in several publications in peer reviewed scientific journals related to the topic of this study and orally in conferences concerning this theme.

## Discussion

Most studies on the effect of SMN-augmenting genetic therapies on respiratory outcomes, do not show significant improvement in lung function parameters in patients with SMA types 2 and 3 [13–16]. Respiratory muscle training (RMT) has been shown effective in patients with pulmonary diseases, kyphoscoliosis, or DMD [23–26]. There are two studies who included patients with SMA, however the groups of patients with SMA were small (*n* = 9, 33% of total number of patients [17] and *n* = 3, 37% of total number of patients [27]) and distinction was not made between the results for the DMD and SMA patients. None of these patients with SMA received any form of SMN-augmenting therapy. To further study the efficacy of a 4-month home-based RMT program in patients with SMA, we designed a randomized controlled trial.

The diaphragm acts as the primary inspiratory muscle and accounts for 70% of the inspired air volume during regular breathing [54]. In patients with SMA, intercostal respiratory muscles are weak while the diaphragm is relatively spared [55, 56] resulting in lower expiratory muscle strength (PEmax) compared to inspiratory muscle strength (PImax) [31]. Therefore, RMT may perhaps be expected to particularly benefit rescue of expiratory muscle function. Here, however, we chose PImax as the primary outcome measure for the following specific reason: it turned out that reference values to identify patients with respiratory muscle weakness and calculate the required sample size are only available for this particular outcome measure (i.e., (PImax ≤ 80 cmH2O; [32]). As a result, it may be difficult to reach the primary endpoint of this study.

Training intensity of at least 30% of PImax and PEmax are necessary to increase the strength of respectively the inspiratory and expiratory muscles [30]. RMT has been studied before in patients with neuromuscular diseases [30, 57], however almost all these studies were conducted in patients with Amyotrophic Lateral Sclerosis (ALS) or myopathies, such as DMD [30, 57]. Frequency of training, duration of the interventions and the intensity of the training programs varied considerably [30, 57]. Only few studies included patients with SMA and none of the studies provided separate data for patients with SMA [30]. In a pre-experimental study with eight participants, including three patients with SMA, participants performed inspiratory muscle training twice a day, 5 days a week, 30 breaths per session, for six weeks [27]. Here, we chose to copy the training intensity and frequency used in this study [27].

Studies suggest that an IMT protocol of training twice a day with PImax as guidance of resistance over a period of three to six months can have a positive effect on inspiratory muscle strength in patients with neuromuscular diseases [57]. Patients who are treated with the *SMN2*-splicing modifying drugs Spinraza® or Risdiplam have their follow up visits in our center every four months. To minimize patient burden, we have opted to combine visits for the RESISTANT trial with these therapy follow-up visits, and we have chosen a training period of 4 months.

Lastly, a recent study on fatigability of respiratory muscles in patients with SMA observed that perceived exertion, measured with an OMNI scale, did not correlate with objective exertion [58]. The OMNI scale has only been validated in children and adults during motor activities [59, 60] and may not detect exertion of the respiratory muscles. Here, we have therefore chosen to monitor the response on respiratory muscle loading with experienced intensity of the training and perceived dyspnea measured with a Borg scale [61].

In conclusion, we will conduct a single blinded randomized sham-controlled trial to investigate the feasibility and efficacy of respiratory muscle training in patients with SMA and respiratory muscle weakness. We hypothesize that RMT is feasible and that it will improve inspiratory and expiratory muscle strength.

## Data Availability

Not applicable.

## Abbreviations

ACT: Airway clearance techniques
AE: Adverse events
ALS: Amyotrophic lateral sclerosis
AS: Airstacking
BOCF: Baseline-observation-carried-forward
BP: Bodily pain
cmH2O: Centimeters of water column
DMD: Duchenne muscular dystrophy
EMT: Expiratory muscle training
ERS/ATS: European respiratory society/ American thoracic society
FEV1: Forced expiratory volume in 1 s
(F)VC: (Forced) vital capacity
GDPR: General data protection regulation
GH: General health perceptions
IMT: Inspiratory muscle training
kPa: Kilopascal
M: Month
MCS: Mental component scale
MEDRA: Medical dictionary for regulatory activities
METC: Medical ethics research committee
MH: General mental health
MI-E: Mechanical insufflation-exsufflation
MRC: Medical research council
P0.1: Mouth occlusion pressure at 100 ms during quiet breathing
PCF: Peak cough flow
PCS: Physical component scale
PedsQL: Pediatric quality of life inventory
PEF: Peak expiratory flow
PEmax: Maximum expiratory mouth pressure
PEP: Positive expiratory pressure
PF: Physical functioning
PImax: Maximum inspiratory mouth pressure
RCT: Randomized sham-controlled trial
RE: Role limitations due to emotional problems
RESISTANT: Respiratory Muscle Training in Patients with Spinal Muscular Atrophy
RMT: Respiratory muscle training
RP: Role limitations due to physical health problems
SD: Standard deviation
SF: Social functioning
SF36: 36-Item short form health survey
SMA: Spinal muscular atrophy
SMN: Survival motor neuron
SNIP: Sniff nasal inspiratory pressure
SPIRIT: Standard protocol items: recommendations for interventional trials
UMCU: University medical center Utrecht
VT: Vitality
WMO: Medical Research Involving Human Subjects Act
